# Magnetic resonance angiography and perfusion mapping by arterial spin labeling using Fourier transform–based velocity-selective pulse trains: Examination on a commercial perfusion phantom

**DOI:** 10.1002/mrm.28805

**Published:** 2021-05-02

**Authors:** Feng Xu, Dan Zhu, Hongli Fan, Hanzhang Lu, Dapeng Liu, Wenbo Li, Qin Qin

**Affiliations:** 1 The Russell H. Morgan Department of Radiology and Radiological Science, Johns Hopkins University, Baltimore, Maryland, USA; 2 F.M. Kirby Research Center for Functional Brain Imaging, Kennedy Krieger Institute, Baltimore, Maryland, USA; 3 Department of Biomedical Engineering, Johns Hopkins University, Baltimore, Maryland, USA

**Keywords:** arterial spin labeling, Fourier transform–based velocity-selective pulse train, magnetic resonance angiography, phantom, velocity-selective inversion, velocity-selective saturation

## Abstract

**Purpose:**

Benchmarking of flow and perfusion MR techniques on standardized phantoms can facilitate the use of advanced angiography and perfusion-mapping techniques across multiple sites, field strength, and vendors. Here, MRA and perfusion mapping by arterial spin labeling (ASL) using Fourier transform (FT)–based velocity-selective saturation and inversion pulse trains were evaluated on a commercial perfusion phantom.

**Methods:**

The FT velocity-selective saturation–based MRA and FT velocity-selective inversion–based ASL perfusion imaging were compared with time-of-flight and pseudo-continuous ASL at 3 T on the perfusion phantom at two controlled flow rates, 175 mL/min and 350 mL/min. Velocity-selective MRA (VSMRA) and velocity-selective ASL (VSASL) were each performed with three velocity-encoding directions: foot–head, left–right, and oblique 45°. The contrast-to-noise ratio for MRA scans and perfusion-weighted signal, as well as labeling efficiency for ASL methods, were quantified.

**Results:**

On this phantom with feeding tubes having only vertical and transverse flow directions, VSMRA and VSASL exhibited the dependence of velocity-encoding directions. The foot–head-encoded VSMRA and VSASL generated similar signal contrasts as time of flight and pseudo-continuous ASL for the two flow rates, respectively. The oblique 45°–encoded VSMRA yielded more uniform contrast-to-noise ratio across slices than foot–head and left–right-encoded VSMRA scans. The oblique 45°–encoded VSASL elevated labeling efficiency from 0.22–0.68 to 0.82–0.90 through more uniform labeling of the entire feeding tubes.

**Conclusion:**

Both FT velocity-selective saturation–based VSMRA and FT velocity-selective inversion–based VSASL were characterized on a commercial perfusion phantom. Careful selection of velocity-encoding directions along the major vessels is recommended for their applications in various organs.

## 1 | INTRODUCTION

Non-contrast-enhanced MRA and perfusion mapping techniques using arterial spin labeling (ASL) appeal to well-characterized sequence modules, to separate the moving water spins in blood vessels from the stationary ones in tissues. Spatially selective prepulses such as presaturation and pre-inversion on the upstream arterial vessels require careful placement, and are sensitive to slow arterial inflow for elderly subjects or patients with large-vessel stenosis or occlusion, and therefore are limited to small spatial coverage.

To circumvent these restrictions, conventional velocity-selective (VS) pulse trains with velocity-encoding gradients have been designed to saturate the signal of blood flowing above a cutoff velocity (Vc) and preserves the ones of the static tissue, by assuming laminar flow distribution within vessels. These flow-dephasing modules have been used right before acquisitions for 3D black-blood MRI^1–3^ and subtraction-based bright-blood MRA.^4–6^ Velocity-selective ASL (VSASL) used the conventional velocity-selective saturation (VSS) pulse trains in the original implementations for measuring cerebral blood flow^7,8^ and cerebral blood volume.^9–12^ Alternatively, the emerging techniques of Fourier transform (FT)–based VS pulse trains are able to saturate (FT-VSS) or invert (FT-VSI) static tissue while preserving spins flowing above V_C_. The FT-VS pulse trains have been applied to non-subtraction-based VSMRA^13–19^ and VSASL-derived quantitative mapping of cerebral blood flow^20–26^ as well as cerebral blood volume.^27^

Several studies have evaluated the characteristics of various VS pulse trains in simple flow phantoms.^5,28–30^ In-house flow phantoms have been used for MRA applications to mimic specific vascular territories and disease conditions.^31–35^ Basic perfusion phantoms were also constructed for evaluating ASL quantifications.^36–38^ It is well recognized that standardized flow and perfusion phantoms, which provide predictable, reproducible, and quantifiable flow dynamics, would facilitate the use of advanced MRA/ASL techniques across multiple sites, field strength, and vendors.^39,40^ A commercial perfusion phantom QASPER (Quantitative Arterial Spin Labeling Perfusion Reference; Gold Standard Phantoms, London, United Kingdom) was recently introduced.^41^ It has been quantitatively characterized by two common ASL methods: flow-sensitive alternating inversion recovery^41^ and pseudo-continuous ASL (PCASL).^42–45^

To lay the groundwork for future multiplatform evaluations using the standardized QASPER phantom, we aimed to compare FT-VSS-based VSMRA and FT-VSI-based VSASL perfusion mapping with time-of-flight (TOF) MRA and PCASL, respectively, on a 3T scanner. As VSASL can be applied for MRA or other applications, in this work VSASL refers to VSASL perfusion imaging exclusively. The effects of velocity-encoding directions and Vc of FT-VS pulse trains were examined in both angiography and perfusion estimation.

## 2 | METHODS

### 2.1 | Experiments

Experiments were conducted on a 3T clinical scanner (Ingenia; Philips Healthcare, Best, The Netherlands) using the body coil for RF transmission (maximum amplitude = 13.5 mT) and a 32-channel head-only coil for signal reception. The maximum strength of the gradient coil was 45 mT/m, and the maximum slew rate was 200 mT/m/ms.

**FIGURE 1:**
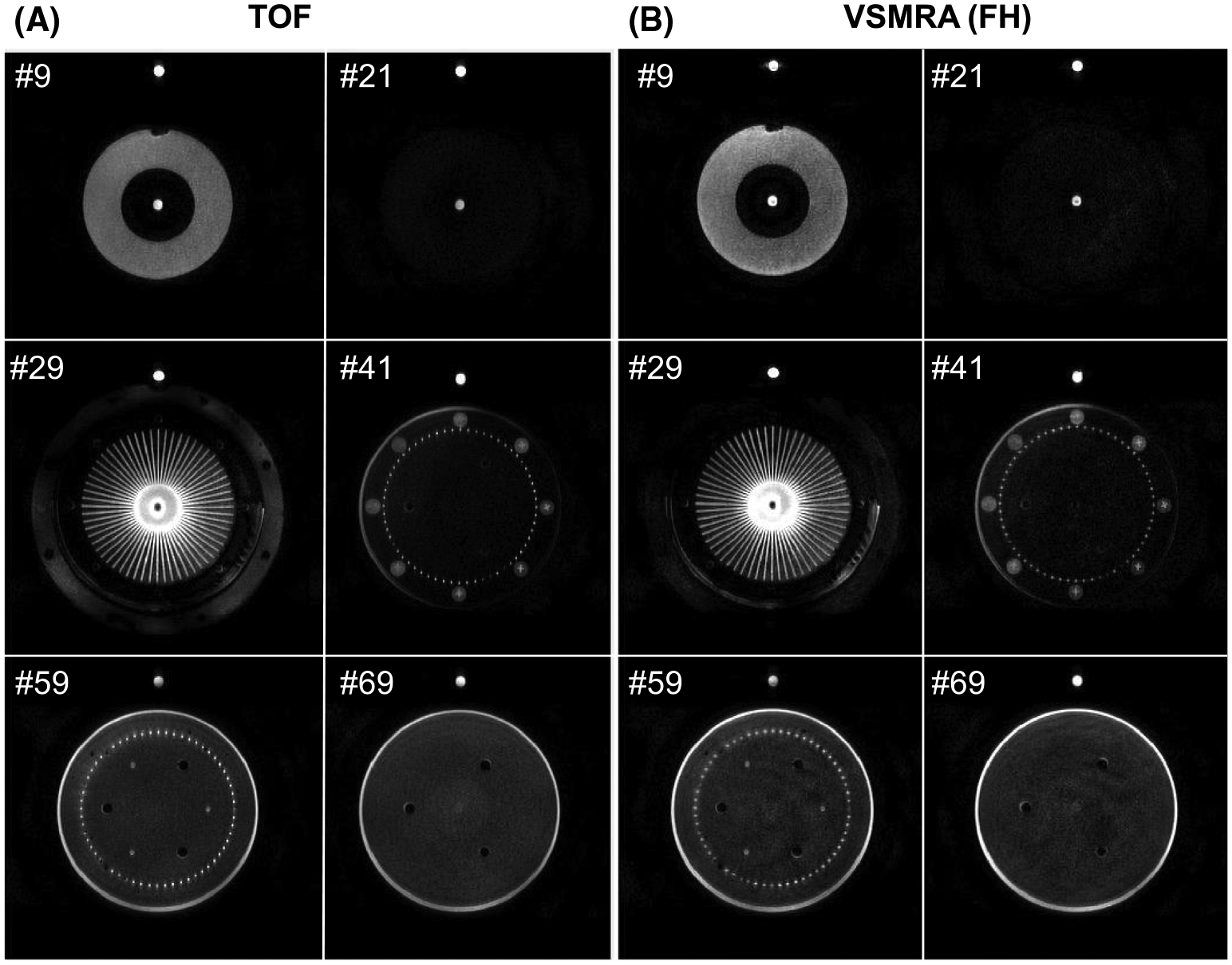
Axial source images of time-of-flight (TOF) (A) and velocity-selective MRA (VSMRA) (B) at six selected slices marked by the yellow lines on the sagittal maximum intensity projection (MIP) of TOF in Supporting Information Figure S1A at 350-mL/min flow rate

The specifics of the QASPER phantom is described online.^46^ The MRA results (detailed subsequently) delineate the structure of the phantom (Supporting Information Figure S1A,B and Figure 1), which contains the main feeding tube within the labeling chamber (for PCASL) and the diverted 60 channels feeding the perfusion chamber (6-cm radius) with a stack of porous material consisting of six perfusion layers.

Two controlled flow rates, first 350 mL/min and then 175 mL/min, were used in this experiment. The 2D phase-contrast MRIs were acquired before and after the MRA and ASL scans to verify the delivered flow. At each flow rate, in addition to TOF and PCASL, FT-VSS-based VSMRA and FT-VSI-based VSASL were each performed with three velocity-encoding directions: foot–head (FH), left–right (LR), and oblique 45° on the coronal plane (O45°) (Supporting Information Figure S1C). All acquisitions were in the transverse orientation. The FT-VS pulse trains used in the current work were applied with 2.0-cm/s Vc. The TOF and VSMRA, as well as PCASL and VSASL, have matched FOV, resolution, and scan time. More details on the MRI protocols are provided in Supporting Information S1.

### 2.2 | Data analysis

For phase-contrast MRI, a region of interest (ROI) of the feeding tube was drawn on the magnitude image and applied to the phase image to compute the mean velocity and the flow rate. Maximum intensity projection images were produced from both TOF and VSMRA scans. Ring ROIs with a thickness of 10 voxels covering 360° were drawn from the MRA raw images to investigate the signal course of the 60 channels right after the diversion in relation to their rotation angles. Two small ring sectors each covering 42° or seven channels were selected at the left and top side of the ring ROIs, to compare their contrast-to-noise ratio for MRA scans. Their contrast-to-noise ratio values were calculated by the mean of the subtractions of the maximum and minimum signal of each channel within the left and top sectors at slice 29, divided by the SD within a centric circle of 24.5-mm radius at slice 59. Similar ring ROIs and left/top sectors were selected on ASL results to compare perfusion-weighted signal (PWS) and labeling efficiency (α) of different ASL methods, with thickness of 4 and 7 voxels for 175 mL/min and 350 mL/min, respectively. The formulas for quantifying PWS and α are provided in Supporting Information S2.

## 3 | RESULTS

### 3.1 | Magnetic resonance angiography experiments

The monitoring system of the perfusion phantom ensured that the flow rate did not alter much during the entire period of MRA and ASL scans at controlled 350 mL/min and 175 mL/min. From the phase-contrast MRI, the measured mean velocities within the cross section of the feeding tube (6-mm diameter) were 18.6/20.3 cm/s and 10.0/9.9 cm/s at the beginning/end of each flow condition, and the measured respective flow rates were 316/344 mL/min and 170/168 mL/min (F = V·π·R^2^, where V is the mean velocity and R is the radius). Accordingly, it was estimated that flowing fluid within the diverted 60 channels (1-mm diameter) had mean velocities of approximately 12 cm/s and 6 cm/s at the two flow rates, and would become slower after entering the perfusion chamber with the porous material and be reduced along a gradient to eventually less than 1 cm/s at the end of these tubes.

In Figure 1, six selected source images of TOF and FH-encoded VSMRA at the controlled 350-mL/min flow rate are shown with their locations marked by the yellow lines on the sagittal maximum intensity projection of TOF (Supporting Information Figure S1A). A more detailed description of the perfusion phantom illustrated in Figure 1 is provided in Supporting Information S3.

**FIGURE 2:**
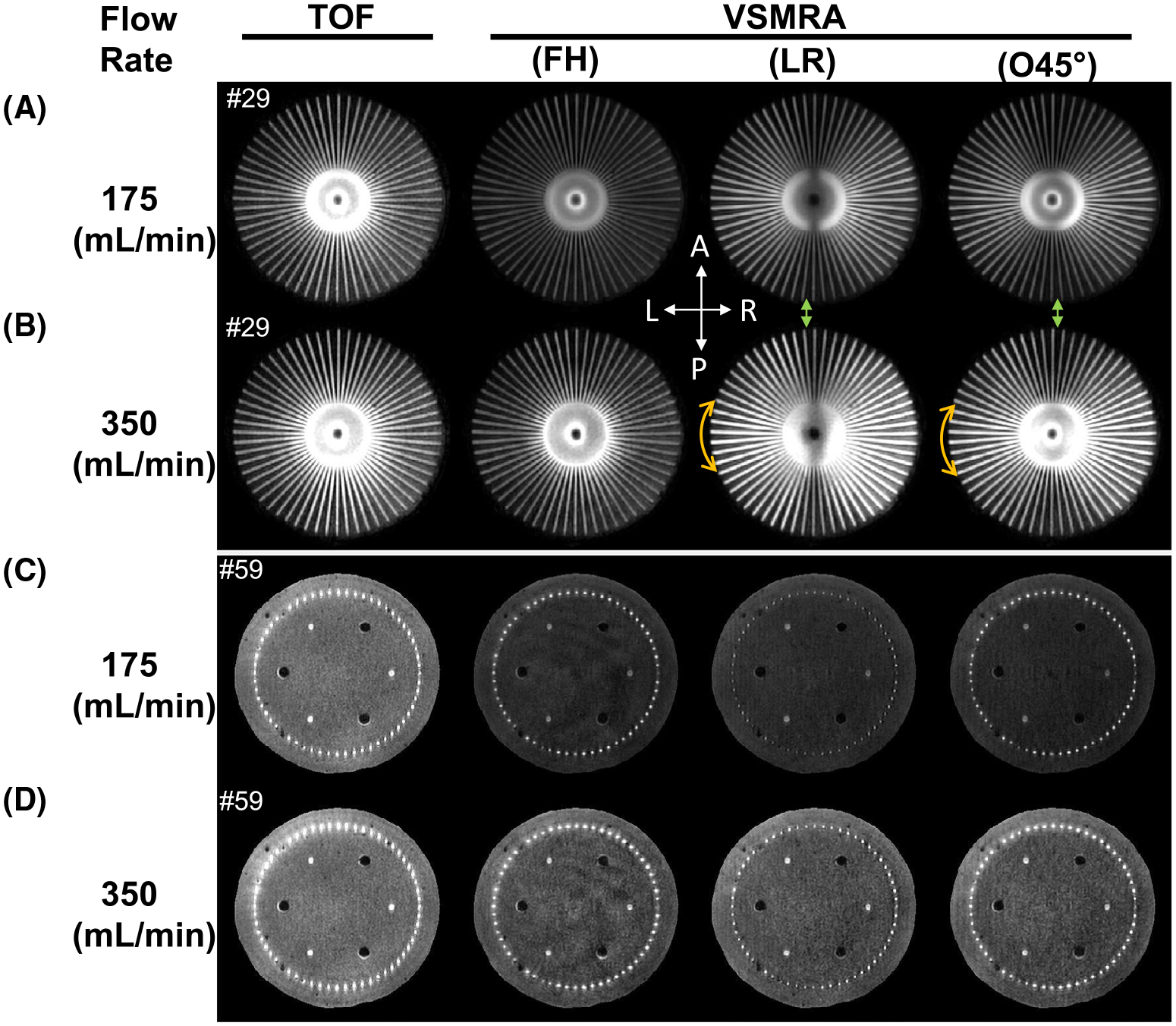
The TOF and VSMRA using foot–head (FH), left–right (LR), and oblique 45° on the coronal plane (O45°) encoded directions at slice 29 (A,B) and slice 59 (C,D) for 175-mL/min (A,C) and 350-mL/min (B,D) flow rates, respectively. The yellow arc of arrows at the left side indicates the higher signal by LR-encoded and O45°-encoded VSMRA than the FH-encoded ones. The green arrows mark the dark bands along the anterior–posterior direction of LR-encoded and O45°-encoded VSMRA. Both reflect poorer signal at the flow directions orthogonal to the velocity-encoding directions

The comparison between TOF and VSMRA with FH, LR, and O45° velocity-encoding directions is shown in Figure 2 at slices 29 and 59 for both 175-mL/min and 350-mL/min flow rates. Velocity-selective MRA revealed its dependence on the velocity-encoding directions. For example, FH encoding yielded lower signal than LR encoding on slice 29 (yellow arc of arrows), whereas it had stronger signal than LR encoding on slice 59. This was because the signal intensity of VSMRA was diminished when its flow direction was orthogonal to the velocity-encoding direction. With encoding the direction of O45° to both FH and LR (Supporting Information Figure S1C), the loss of signal for FH encoding on slice 29 and for LR encoding on slice 59 were both mitigated (see green shades in Supporting Information Figure S2 for more quantitative plots). However, there was a central dark band along the anterior–posterior direction at slice 29 (green arrows) due to its perpendicular direction to both LR and O45°.

Supporting Information Figure S2 arrays the signal course of slice 29 by the four MRA scans from a ring ROI indicated by two red circles in the MRA image, with rotation angles labeled every 45° around the ring. The averaged signal along the radius within the ring ROI as a function of rotation angles ranging from 45° to 225° is displayed for each MRA scan. The maximal and minimal signals across angles were highly correlated, which partly was due to the sensitivity of the receive coils. Both TOF and VSMRA scans at 175 mL/min produced lower signal than the corresponding ones at 350 mL/min. The LR and O45° encodings generated greater signal than FH encoding for most angles. The left and top sectors marked with green and yellow bars at the ring ROI of slice 29 on the raw MRA image are shaded with the same colors at the signal courses of each MRA scan. The contrast-to-noise ratio values from these two small sectors of four MRA scans at two flow conditions are listed in Table 1, with quantitative comparisons provided in Supporting Information S4.

### 3.2 | Arterial spin labeling experiments

**FIGURE 3:**
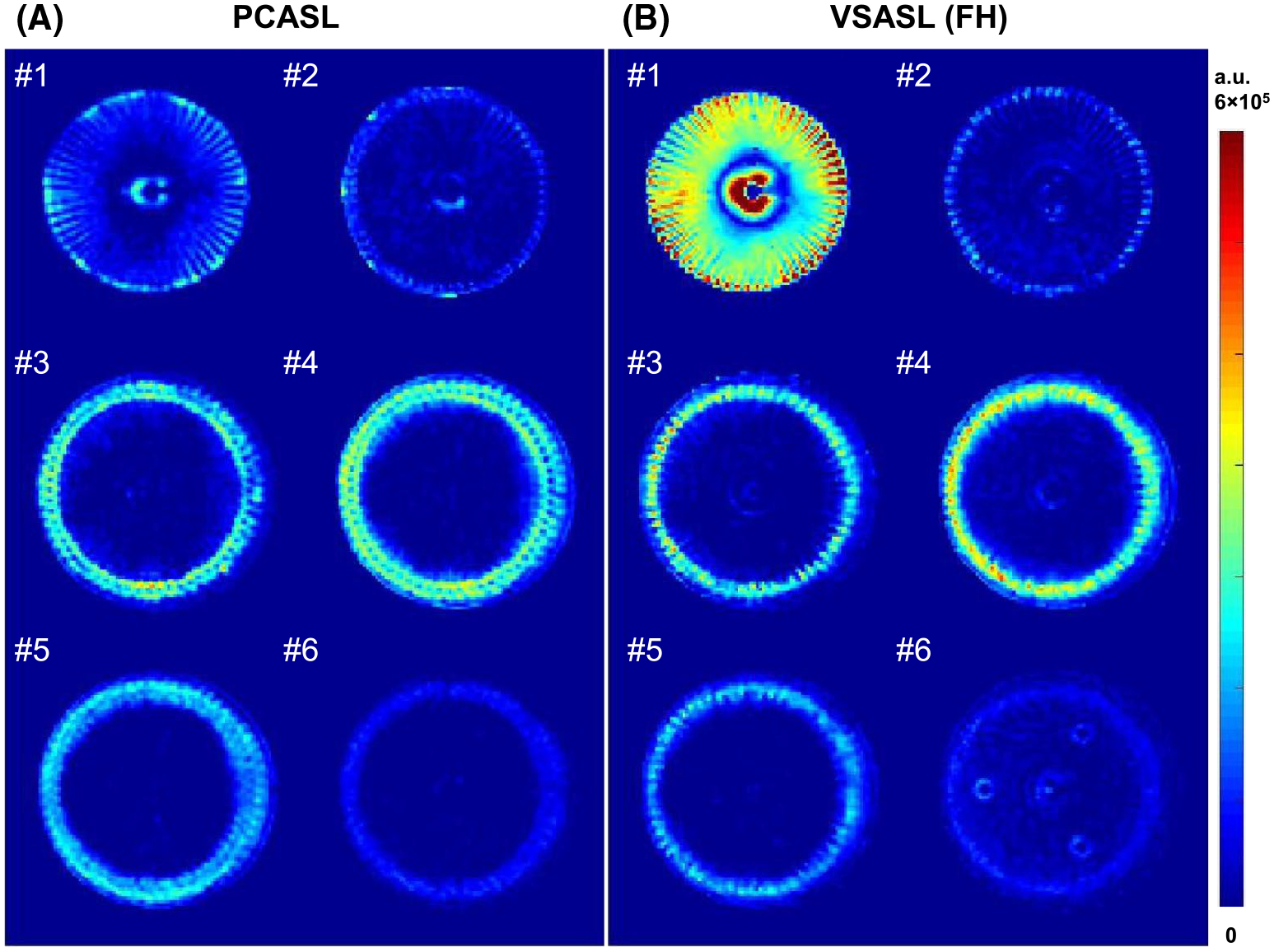
The difference images (subtraction of label and control) of pseudo-continuous arterial spin labeling (PCASL) (A) and velocity-selective arterial spin labeling (VSASL) (B) at the six slices marked by the yellow lines on the sagittal maximum intensity projection (MIP) of VSMRA in Supporting Information Figure S1B at the 350-mL/min flow rate. The VSASL (FH) technique is a velocity-selective inversion-prepared ASL with a foot–head encoding direction. All images are displayed at the same scale

The ASL difference images in the acquired six slices at 350 mL/min are illustrated in Figure 3 for PCASL and VSASL with FH encoding. The 60 channels were enclosed in slice 1 when flowing radially right after the diversion from the feeding tube. They passed through slices 3 and 4 when flowing axially, and discontinued before slice 5. The perfusion layers were located from slices 3 to 6. Visual inspection showed that, at this flow rate, PCASL and the FH-encoded VSASL generated largely comparable contrast patterns at the perfusion layers. The VSASL method produced more difference signal than PCASL within the 60 channels in slices 1, 3, and 4. This indicated that the bolus duration of VSASL was longer than the postlabeling delay (1.5 seconds), which was further exemplified when the flow crusher was switched off.

Supporting Information Figure S3 compares the crusher effect at slice 4 for both PCASL and VSASL at the two flow conditions. The VSASL method without the crusher yielded considerably more difference signal inside the 60 channels than VSASL with the crusher for both 175-mL/min and 350-mL/min flow rates. In contrast, much smaller differences were seen for PCASL inside the 60 channels, as the bolus duration of PCASL was set equal to its postlabeling delay (1.8 seconds), which was long enough to allow most bolus to leave the channels and enter the porous material. For the flow rate of 175 mL/min, when transit time was longer due to the slower velocity, both PCASL and VSASL delivered less expansion of perfusion signal than at the 350-mL/min flow rate.

**FIGURE 4:**
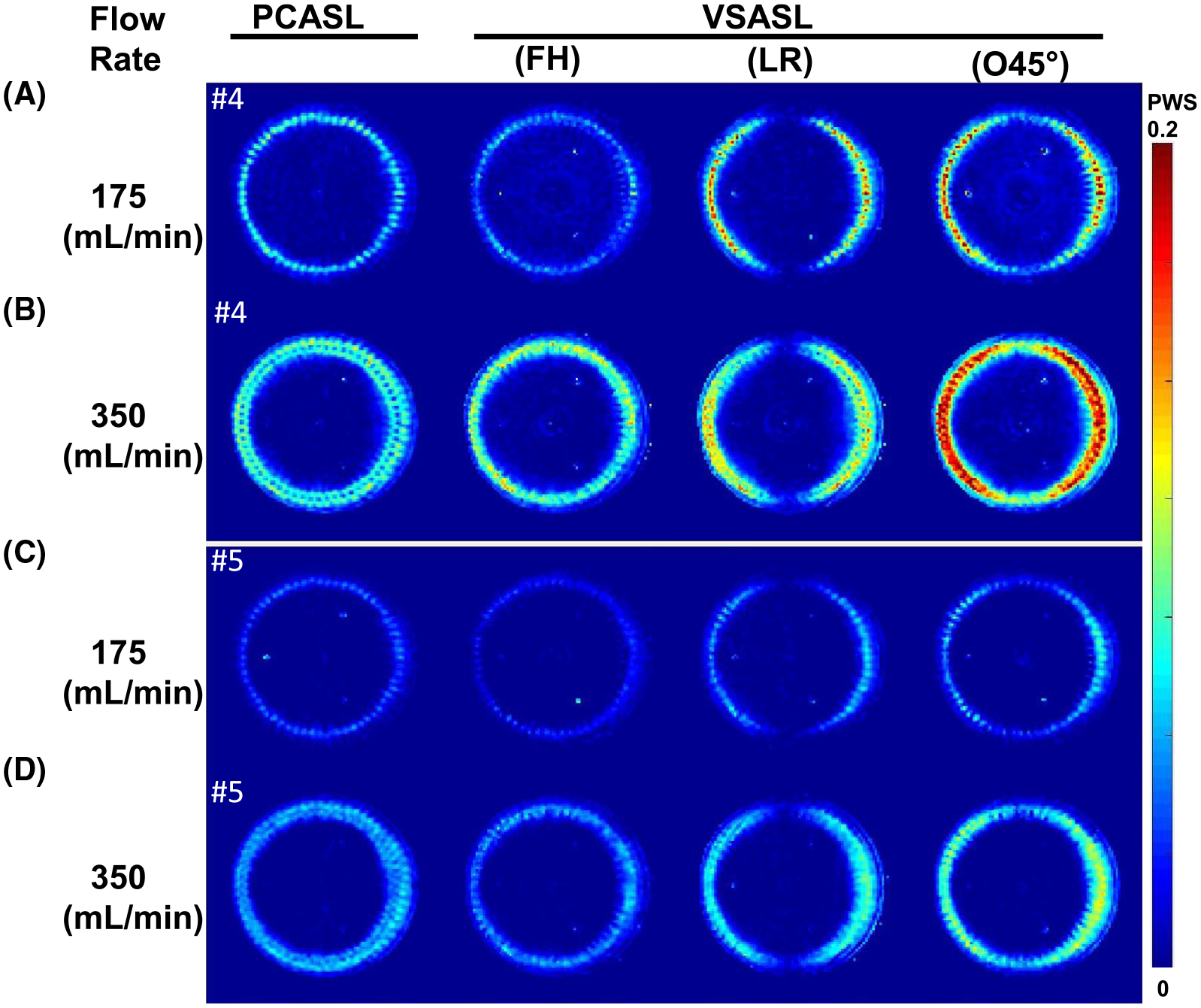
The perfusion-weighted signal (PWS: difference signal normalized by the SI_PD_) of PCASL and VSASL using FH, LR, and O45°-encoded directions at slice 4 (A,B) and slice 5 (C,D) for 175-mL/min (A,C) and 350-mL/min (B,D) flow rates, respectively

The PWS images of PCASL and VSASL with encoding directions of FH, LR, and O45° at two flow rates are shown in Figure 4 for both slices 4 and 5. Similar to Supporting Information Figure S3, FH-encoded VSASL produced much lower PWS throughout the disk at 175 mL/min than at 350 mL/min. This can be understood, as the perfusion phantom has a large portion of the bolus located at the 60 radial channels before entering the perfusion layers, and this transverse plane is perpendicular to the FH-encoding direction. Conversely, LR-encoded VSASL demonstrated higher perfusion signal at both the left and right sides. The VSASL method with O45° encoding combined labeling bolus in both FH and LR encoding, thus yielding strongest perfusion signal at bilateral sides. As in VSMRA (Figure 2A,B), both LR-encoded and O45°-encoded VSASL also resulted in central dark bands along the anterior–posterior direction.

Supporting Information Figure S4 displays the PWS of each ASL scan as a function of rotation angles at slice 5 from their ring ROIs. Like MRA signal courses in Supporting Information Figure S2, LR and O45° signals dipped at the top sector, reflecting their direction dependence of the labeling preparation. By setting the PCASL-derived cerebral blood flow values averaged between the two sectors at 350 mL/min (calculated as 144 mL/100g/min based on Eq. 2 in Supporting Information S2) as a reference (half of cerebral blood flow for 175 mL/min), the labeling efficiency for different methods were estimated using Eqs. 4 and 5 in Supporting Information S2. Averaged PWS and labeling efficiency from the left and top sectors are listed in Table 1, with quantitative comparisons provided in Supporting Information S5. Among the VSASL methods, LR-encoded and O45°-encoded scans for the left sector had the higher labeling efficiency (0.90 and 0.90 for the 175-mL/min flow rate, and 0.68 and 0.82 for the 350-mL/min flow rate).

## 4 | DISCUSSION

The VSMRA and VSASL methods based on FT-VS pulse trains were examined on a commercially available flow/perfusion phantom. At two controlled flow rates and with 60 diverted channels through different flow paths, the dependence of velocity-encoding directions of VSMRA and VSASL were characterized by comparing them with TOF and PCASL, respectively. When the encoding direction was aligned with the flow path, VSMRA and VSASL delivered the best performance and yielded better results than TOF and PCASL.

Unlike the curved vessels commonly observed in vivo, the QASPER phantom has the fluid flow axially first, then split into the 60 diverted channels in radial directions within a transverse plane, and later turned axially again. Under the flow rates of 175 mL/min or 350 mL/min, the mean velocities of fluid reduced from 10 cm/s or 20 cm/s in the feeding tube to 6 cm/s or 12 cm/s in the 60 channels with different angular rotations; the corresponding mean transit times were 1.0 second or 0.5 second through the channels within the transverse plane (6-cm length) and 0.33 second or 0.17 second through the channels in the perfusion layers (4 layers • 0.5 cm = 2-cm length).

With Vc = 2 cm/s, the FH-encoded VSMRA scan generated similar results as TOF, with the signal in the feeding tube as well as the later portion of the 60 channels (after turning vertically) higher than the signal in the beginning portion of the 60 channels (Figure 1 and Supporting Information Figures S1 and S2). For LR-encoded VSMRA, the channels along the LR direction in the transverse plane as well as the ones within ±70° or ±80° angles to LR direction are well-depicted for the two flow rates (Figure 2), as their projected velocities along LR direction were larger than Vc (ie, 6·cos[70°] = 2.1 cm/s and 12·cos[80°] = 2.1 cm/s). The O45°-encoded VSMRA yielded more uniform signal between the channels close to the LR direction in the transverse plane and the ones running axially (Figure 2). In contrast, a small number of channels along the anterior–posterior directions in the transverse plane showed relatively lower signal (the dark band) under LR and O45°-encoded VSMRA (Figure 2 and Supporting Information Figure S2). These observations were supported by the quantitative contrast-to-noise ratio analysis (Table 1).

The FH-encoded VSI-prepared ASL displayed similar signal contrast as PCASL inside the perfusion chamber for the two flow rates (Figure 3 and Supporting Information Figures S3 and S4). As mentioned previously, the remaining signal within the beginning portion of the 60 channels in the transverse plane by VSASL reflected its bolus duration longer than the postlabeling delay used (Figure 3B). This large-vessel effect was more visible at 175 mL/min, as slower velocity led to longer bolus, and it was considerably mitigated by the crusher (Supporting Information Figure S3). Compared with PCASL, the PWS by FH-encoded VSASL was 52% lower at 175 mL/min and 31% lower at 350 mL/min (Table 1). This difference could be explained by the larger fraction of the bolus duration originating from channels in the transverse plane at the lower flow rate: 67% (transit time/postlabeling delay = 1.0/1.5) versus 33% (0.5/1.5). Note that the contribution of the bolus duration originating from channels in the perfusion layers was even smaller: 22% (0.33/1.5) and 11% (0.17/1.5). Thus, LR-encoded VSASL gained more PWS: 70% higher at 175 mL/min versus 25% higher at 350 mL/min than PCASL (Table 1). With more uniform labeling over the bolus within channels of both FH and LR directions (Figure 2), the O45°-encoded VSASL generated higher PWS than other ASL methods (Figure 4 and Table 1), resulting in its labeling efficiency elevated from 0.22–0.68 to 0.82–0.90 (Table 1).

Careful selection of velocity-encoding directions along the major vessels is recommended for applications in various organs. The special configuration of QASPER phantom amplifies this issue. For abdominal VSMRA, O45° encoding was chosen to cover both the descending aorta and renal arteries.^19^ The direction dependence of conventional VS pulse trains has also been demonstrated for a phantom and the hand MRA,^5^ as well as the brain VSASL for larger values of Vc.^8^ When Vc is below 4 cm/s, previous work on cerebral VSMRA and VSASL showed much improved isotropy with respect to velocity-encoding directions.^8,15^ Realistically, there would always be some vessels in a plane perpendicular to the selected direction. Thus, the labeling efficiency for VSASL would be uniformly degraded with different velocity-encoding directions. Furthermore, lowering Vc could potentially mitigate this challenge, as it requires higher gradient strength and longer pulse train duration. Note that the high labeling efficiency (0.82–0.90) achieved by O45°-encoded VSASL in this phantom study would not be realized in vivo by only choosing the optimal directions alone, as there are other sources for signal loss of FT-based VS pulse trains, such as T_2_ relaxation, flow acceleration, and B_0_/B_1_^+^ field in-homogeneities.^20,23,26^ Nonetheless, reducing this direction dependence on both VSMRA and VSASL would facilitate their clinical adoptions.

## 5 | CONCLUSIONS

Both FT-VSS-based VSMRA and FT-VSI-based VSASL were evaluated on a commercial flow/perfusion phantom. With the characterization reported here, the two methods could be evaluated on the same phantom systematically at any scanner elsewhere. This study is expected to facilitate future technical development, validation, and quality assurance. The standardized assessment would ensure accuracy and repeatability for angiography and perfusion measurements in wider clinical applications.

## Supplementary Material

1 **FIGURE S1** Sagittal maximum intensity projection (MIP) of the perfusion phantom obtained by time-of-flight (TOF) (A) and velocity-selective MRA (VSMRA) (B) at a flow rate of 350 mL/min. The yellow lines on TOF indicate the slice location of the MRA images. The yellow lines on VSMRA represent slice locations of the ASL images. (C) Three velocity-encoding directions, foot–head (FH), left–right (LR), and oblique 45° on the coronal plane (O45°), were used in VSMRA and velocity-selective arterial spin labeling (VSASL) scans in this study with the same cut-off velocity
**FIGURE S2** The signal course within a ring region of interest (ROI) as a function of rotation angles is displayed for TOF and VSMRA with three velocity-encoding directions (A) at 175 mL/min (B) and 350 mL/min (C) flow rates. A, The ring ROI is enclosed by two red circles in the MRA image of slice 29, labeled every 45° around the disk. The left and top sectors were marked with green and yellow bars at the ring ROI (A) and shaded with the same colors at the signal course under each MRA scan (B,C)
**FIGURE S3** Difference images of PCASL and FH-encoded VSASL at slice 4 with and without the crusher module at 175 mL/min (A) and 350 mL/min (B)
**FIGURE S4** The perfusion-weighted signal (PWS: difference signal normalized by the SI_PD_) within a ring ROI as a function of rotation angles is displayed for PCASL and VSASL with three velocity-encoding directions at 175 mL/min (A,C) and 350 mL/min (B,D) flow rates. A,B, The ring ROIs are enclosed by two red circles in the ASL images of slice 5 for each flow rate, respectively. The left and top sectors are marked with green and yellow bars at the ring ROIs (A,B) and shaded with the same colors at the signal course under each ASL scan (C,D)

## Figures and Tables

**TABLE 1:** Contrast-to-noise ratio by TOF and VSMRA as well as PWS and labeling efficiency of PCASL and VSASL with three velocity-encoding directions at the left and top sectors for two flow rates

Flow rate (mL/min)	Sector	CNR				PWS				Labeling efficiency			
			VSMRA				VSASL				VSASL		
		TOF	FH	LR	O45°	PCASL	FH	LR	O45°	PCASL	FH	LR	O45°
175	Left	17.3	16.5	42.3	39.2	2.3%	1.1%	3.9%	3.9%	0.83	0.25	0.90	0.90
	Top	23.0	23.5	31.7	29.7	2.9%	1.8%	1.2%	2.2%		0.33	0.22	0.40
350	Left	18.9	12.5	32.5	28.3	5.1%	3.5%	6.4%	7.7%	0.85	0.37	0.68	0.82
	Top	26.0	17.1	24.9	20.0	5.6%	4.6%	2.8%	5.4%		0.45	0.27	0.53

*Note:* The MRA data are from slice 29; ASL data are from slice 5.

Abbreviation: CNR, contrast-to-noise ratio.
