# Supplementary material for: Magnetic resonance angiography and perfusion mapping by arterial spin labeling using Fourier transform–based velocity-selective pulse trains: Examination on a commercial perfusion phantom
Source: Magn Reson Med. Author manuscript; Available in PMC 2022 Feb 22. (PMC8861891; doi:10.1002/mrm.28805)

Supporting Information S1: The detailed parameters of MRI protocols.

FT-VS Pulse Trains

The FT-VS pulse trains used in the current work were comprised of a series of nine excitation pulses ($10°_x$ hard pulses for FT-VSS and $20°_x$ hard pulses for FT-VSI), interleaved with paired and phase-cycled refocusing pulses ($90°_x180°_y90°_x$ composite pulses) (1). Refocusing pulses were surrounded by gradient lobes with alternating polarity (23 mT/m, 0.6 ms duration, 0.2 ms ramp time). Note that for encoding along O45° direction (Figure 1c), the gradients were applied on both FH and LR directions with the strength of 16.3 mT/m. Each velocity-encoding step was 8 ms and the total duration T_{VS} was 64 ms. These gradient configurations were set to correspond to the 2.0 cm/s Vc (the cutoff velocity: below which the slow-moving spins are saturated or inverted and above which the fast-moving spins are preserved). Vc was calculated using the same equation as applied for the conventional VSS pulse trains, $Vc = \pi/(\gamma m_1)$, where γ is the proton gyromagnetic ratio and m_1 is the applied first gradient moment. For VSASL, the control module of the FT-VSI pulse train utilized gradient lobes with the same polarity (velocity-compensated, $m_1 = 0$) (1,2).

MRA Sequences

PC MRIs used a single slice placed perpendicular to the feeding tube 55 mm below the center of imaging volume. The parameters of PC MRI acquisition were: slice thickness = 5 mm, FOV = 180 x 180 mm^2, acquired resolution = 1.15 x 1.15 mm^2, reconstructed resolution = 0.56 x 0.56 mm^2, TR/ TE = 8.9/5.3 ms, flip angle = 10°, maximum encoded velocity along FH direction = 40 cm/s.

For VSMRA, the acquisition module was applied immediately (10 ms gap) after the FT-VSS module using a 3D segmented turbo field echo (TFE) readout with flow compensation and low-high profile ordering (3). The acquisition parameters were: FOV = 180 x 180 x 40 mm^3, acquired resolution = 0.7 x 0.7 x 1.0 mm^3, reconstructed resolution = 0.35 x 0.35 x 0.5 mm^3, TR/TE = 9.1/2.6 ms, flip angle = 14°, TFE factor = 44, acquisition window = 400 ms, readout bandwidth = 108 Hz/pixel, compressed sensing SENSE factor = 2, interval between TFE shots = 2000 ms, number of TFE shots = 93, total scan duration = 3 min.

TOF matched VSMRA for FOV, resolution and scan time. Other parameters were: TR/TE = 22/2.6 ms, flip angle = 18°, readout bandwidth = 134 Hz/pixel, compressed sensing SENSE factor = 1.7, four chunks with 10 slices per chunk.

ASL Sequences

The ASL pulse sequences include a slab-selective pre-saturation at the imaging volume, the label/control modules using either pseudo-continuous or FT-VSI pulse trains, post labeling delay (PLD), a VSS pulse train to crush fast-flowing signal, and a 3D GRASE readout. Background suppression pulses were omitted for this phantom study as there is no physiological noise or motion effect from the static signal. Spatially selective pre-saturation was a product module ("WET") with three slab-selective saturation pulses consecutively applied. The flow-dephasing VSS module used a T_2 prepare pulse train (TE_{prep} = 20 ms) comprising ±90° hard pulses and double refocused composite pulses with two pairs of gradient lobes to suppress the spins flowing above 3.0 cm/s in the FH direction. Comparisons were also made between ASL scans with and without this flow crusher.

For VSI-ASL (1,2), there was a 3 s delay between the pre-saturation and the labeling modules to allow the inflow of fresh solvent into the perfusion chamber. The PLD of FT-VSI was set as 1.5 s. PCASL used labeling duration τ = 1.8 s and PLD = 1.8 s as recommended in the ASL white paper (4). The labeling plane was about 60 mm below the center of the imaging volume.

The acquisition parameters were the same for VSASL and PCASL: 3D GRASE, FOV = 180 x 180 x 30 mm^3, acquired resolution = 1.8 x 1.9 x 5.0 mm^3, reconstructed resolution = 1.4 x 1.4 x 5.0 mm^3, 6 slices, EPI factor = 15, SENSE factor = 2 along the phase-encoding direction, turbo spin echo (TSE) factor = 6 along the slice direction, duration of acquisition window = 109 ms to control blurring and maintain high SNR efficiency (5), TE = 18 ms, and TR = 4.8/5.0 s for VSASL/PCASL. Three pairs of label and control scans were acquired with a total duration of 3.5 min. A proton density-weighted image (SI_{PD}) scan with the same acquisition parameters but TR of 10 s was performed at each flow rate for perfusion quantification.

Supporting Information S2: Equations for quantifying perfusion-weighted signal and labeling efficiency of ASL data.

The perfusion-weighted signal (PWS) was defined as the difference signal (ΔSI) normalized by the SI_{PD}:

$$\mathrm{PWS} = \frac{\Delta \mathrm{SI}}{\mathrm{SI}_{PD}} \qquad [1]$$

For PCASL the CBF was calculated based on the standard single-compartment model (4):

$$CBF = \frac{6000 \cdot \lambda \cdot PWS_{PCASL} \cdot e^{\frac{PLD_{PCASL}}{\mathrm{T}_1}}}{2 \cdot \alpha_{PCASL} \cdot T_1 \cdot (1 - e^{-\frac{\tau_{PCASL}}{T_1}})} \qquad [2]$$

where α_{PCASL} = 0.85 (4); PLD_{PCASL} = 1.8 s; τ_{PCASL} = 1.8 s. Here T_1 of the fluid = 1.8 s and void volume fraction λ = 0.32 in the porous material (6).

For VSI-ASL, the calculation was as given previously (1,2):

$$CBF = \frac{6000 \cdot \lambda \cdot PWS_{VSASL} \cdot e^{\frac{PLD_{VSASL}}{\mathrm{T}_1}}}{2 \cdot \alpha_{VSASL} \cdot PLD_{VSASL}} \qquad [3]$$

where PLD_{VSASL} = 1.5 s. As the CBF values were assumed to be equal between the two methods for the same flow rate, α_{VSASL} was determined as:

$$\alpha_{VSASL} = \frac{PWS_{VSASL}}{PWS_{PCASL}} \cdot \mathrm{C}_{ratio} \cdot \alpha_{PCASL} \qquad [4]$$

Here this constant ratio (C_{ratio}) was derived from Eq. [2] and [3]:

$$\mathrm{C}_{ratio} = e^{\frac{PLD_{VSASL} - PLD_{PCASL}}{\mathrm{T}_1}} \cdot \frac{T_1 \cdot (1 - e^{-\frac{\tau_{PCASL}}{T_1}})}{PLD_{VSASL}} \qquad [5]$$

Based on the values provided above, C_{ratio} = 0.64.

Note that the impulse response function of this standard single-compartment model (4) only considers a magnetization relaxation function $e^{-t/T1}$, and ignores a flow clearance function

$e^{-ft/\lambda}$ (7). For the phantom at flow rate of 350 mL/min, using the CBF of 144 mL/100g/min (from the PCASL Results using Eq. [2]) or f = 144/6000 mL/g/s and $\lambda = 0.32$, f/λ is approximately 13.5% of $1/T_1$. In this case, two-compartment kinetic models that account for this flow clearance function would yield more accurate quantification of phantom CBF. This analysis would inevitably become more complicated and thus exceed the scope of this study. As both PCASL and VSASL applied the same assumption here, the comparison between the two methods are still largely valid.

Supporting Information S3: More description of the perfusion phantom illustrated in Figure 1.

In slices #9 and #21, the central bright cylinder is the feeding tube, while the acentric one is the draining tube from the top of the phantom. The ring surrounding the feeding tube (slices #9) is the water container, and was masked out on sagittal MIP images (Supporting Information Figure S1a,b) for better visualization of the flow inside the perfusion phantom. In slice #29, the feeding tube diverts into 60 transverse radial channels, flowing perpendicularly to the main axis of the phantom with a length of 58 mm before turning 90 degrees and flowing axially (off-center) again into the perfusion chamber. The 60 channels extend for the first two perfusion layers of about 10 mm thickness (slice #41 and #59), then stopped before the 3rd layer (slice #69). Therefore, the 60 bright dots in a ring are visible on slices #41 and #59 but not on slice #69.

Supporting Information S4: Quantitative comparisons of the CNR values of different MRA scans listed in Table 1.

At the left sector, the CNR by FH-encoded VSMRA was comparable to the CNR by TOF at 175 mL/min and 33% lower at 350 mL/min; while the LR and O45° encoded VSMRA delivered 156%, 138% higher CNR at 175 mL/min and 160%, 126% higher at 350 mL/min when compared to FH-encoded VSMRA, and 145%, 127% higher CNR at 175 mL/min and 72%, 50% higher at 350 mL/min when compared to TOF. For TOF and FH-encoded VSMRA, the CNR values at the left sector were 25-30% lower than the CNR at the top sector at the two flow rates. In contrast, for LR and O45° encoded VSMRA, the CNR values at the left sector were 31-42% higher than the ones at the top sector. Between the two flow rates, the CNR of TOF were comparable for both left and top sectors; while VSMRA at 350 mL/min yielded CNR values 18-31% lower than the corresponding ones at 175 mL/min. The discrepancy of the latter was a result of the almost twice of standard deviation values at 350 vs 175 mL/min (data not shown), which could be explained by the slow motion of the fluid within the perfusion chamber.

Supporting Information S5: Quantitative comparisons of the perfusion-weighted signal (PWS) and labeling efficiency values of different ASL scans listed in Table 1.

At the left sector, the PWS by FH-encoded VSASL was 52% lower than the PWS by PCASL at 175 mL/min and 31% lower at 350 mL/min; while the LR and O45° encoded VSASL delivered 70% higher PWS than PCASL at 175 mL/min and 25%, 51% higher at 350 mL/min. For PCASL and FH-encoded VSASL, the PWS at the left sector were 21%, 9% and 39%, 24% lower than the PWS at the top sector for 175, 350 mL/min respectively. In contrast, for LR- and O45°-encoded VSASL, the PWS at the left sector were 225%, 129% and 77%, 43% higher than the PWS at the top sector for 175, 350 mL/min respectively. For twice of the controlled flow rate, PWS at the left sector of PCASL and O45°-encoded VSASL under 350 mL/min flow rate were 222% and 197% of their PWS under 175 mL/min.

Reference

1. Liu D, Xu F, Li W, van Zijl PC, Lin DD, Qin Q. Improved velocity-selective-inversion arterial spin labeling for cerebral blood flow mapping with 3D acquisition. Magn Reson Med 2020;84:2512-2522.
2. Qin Q, van Zijl PC. Velocity-selective-inversion prepared arterial spin labeling. Magn Reson Med 2016;76(4):1136-1148.
3. Qin Q, Shin T, Schär M, Guo H, Chen H, Qiao Y. Velocity-selective magnetization-prepared non-contrast-enhanced cerebral MR angiography at 3 Tesla: Improved immunity to B0/B1 inhomogeneity. Magn Reson Med 2016;75(3):1232-1241.
4. Alsop DC, Detre JA, Golay X, Gunther M, Hendrikse J, Hernandez-Garcia L, Lu H, Macintosh BJ, Parkes LM, Smits M, van Osch MJ, Wang DJ, Wong EC, Zaharchuk G. Recommended implementation of arterial spin-labeled perfusion MRI for clinical applications: A consensus of the ISMRM perfusion study group and the European consortium for ASL in dementia. Magn Reson Med 2015;73:102-116.
5. Qin Q. Point spread functions of the T(2) decay in k-space trajectories with long echo train. Magnetic resonance imaging 2012;30(8):1134-1142.
6. Oliver-Taylor A, Hampshire T, Mutsaerts HJ, Clement P, Warnert E, Kuijer JPA, Baas K, Petr J, Siero JCW, Marques JP, Sunaert S, Borra RJH, van Osch MJP, Golay X, Achten E. A multi-site round robin assessment of ASL using a perfusion phantom. ISMRM. Montréal, QC, Canada. 2019. p 2653.
7. Buxton RB, Frank LR, Wong EC, Siewert B, Warach S, Edelman RR. A general kinetic model for quantitative perfusion imaging with arterial spin labeling. Magn Reson Med 1998;40(3):383-396.

Supporting Information Figure S1: Sagittal maximum-intensity-projection (MIP) of the perfusion phantom obtained by (a) TOF and (b) VSMRA at 350 mL/min flow rate. VSMRA (FH) is velocity-selective saturation prepared MRA with a foot-head encoding direction. Yellow lines on TOF indicate the slice location of following MRA images. Yellow lines on VSMRA represent slice locations of following ASL images. (c) Three velocity-encoding directions, foot-head (FH), left-right (LR), and oblique 45° on the coronal plane (O45°), were used in VSMRA and VSASL scans in this study with the same cut-off velocity.

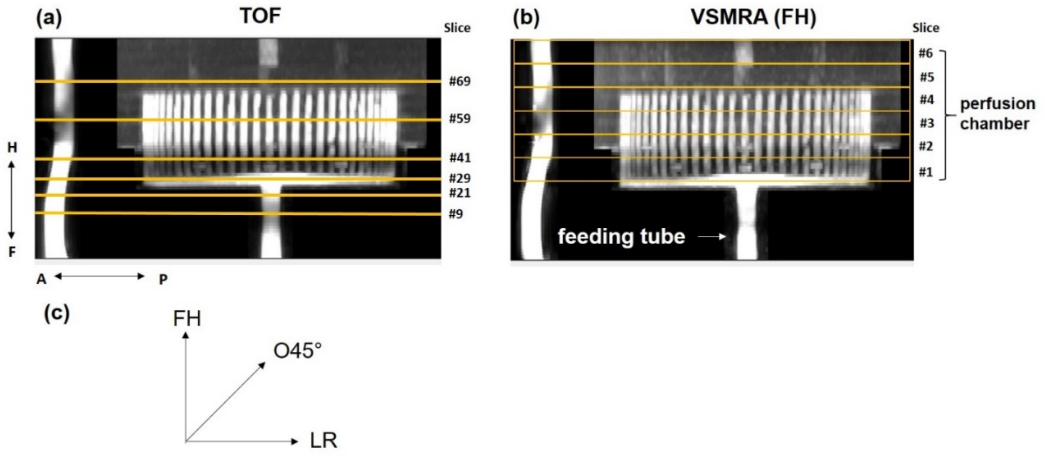

Supporting Information Figure S2: The signal course within (a) a ring ROI as a function of rotation angles were displayed for TOF and VSMRA with three velocity-encoding directions at (b) 175 and (c) 350 mL/min flow rates. (a) The ring ROI is enclosed by two red circles in the MRA image of slice #29 labeled every 45° around the disk. The left and top sectors were (a) marked with green and yellow bars at the ring ROI and (b, c) shaded with the same colors at the signal course under each MRA scan.

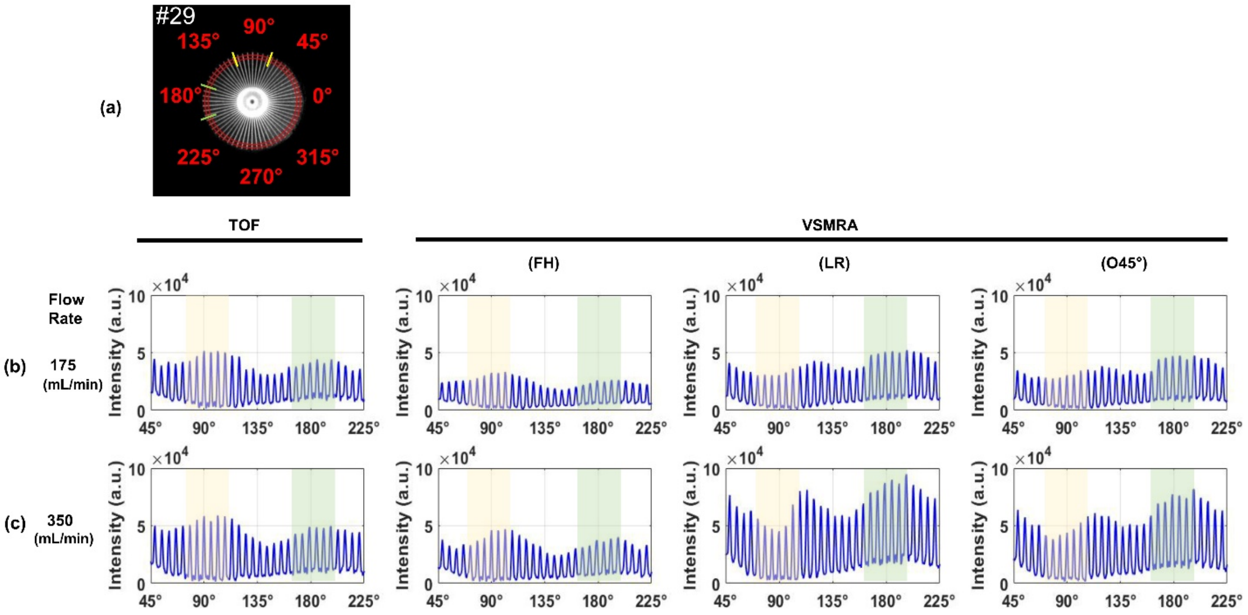

Supporting Information Figure S3: The difference images of PCASL and FH-encoded VSASL at slice #4 with and without the crusher module at (a)175 and (b) 350 mL/min.

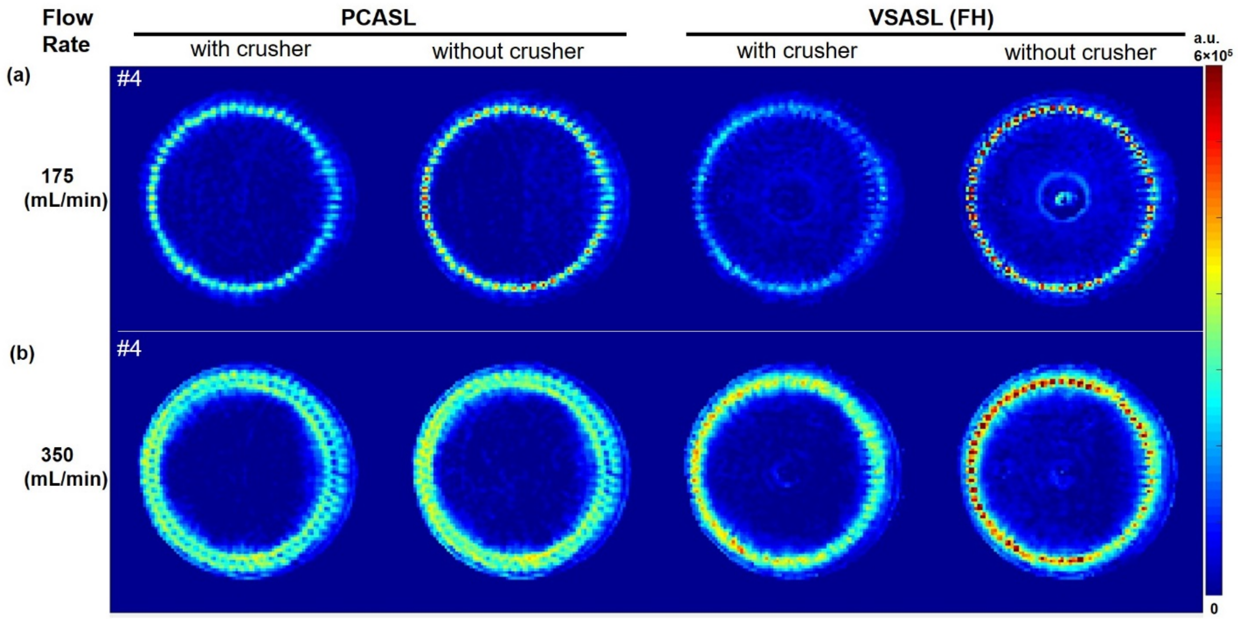

Supporting Information Figure S4: The perfusion-weighted signal (PWS: difference signal normalized by the SI_{PD}) within a ring ROI as a function of rotation angles were displayed for PCASL and VSASL with three velocity-encoding directions at (a, c) 175 and (b, d) 350 mL/min flow rates. (a, b) The ring ROIs are enclosed by two red circles in the ASL images of slice #5 for each flow rate respectively. The left and top sectors were (a, b) marked with green and yellow bars at the ring ROIs and (c, d) shaded with the same colors at the signal course under each ASL scan.

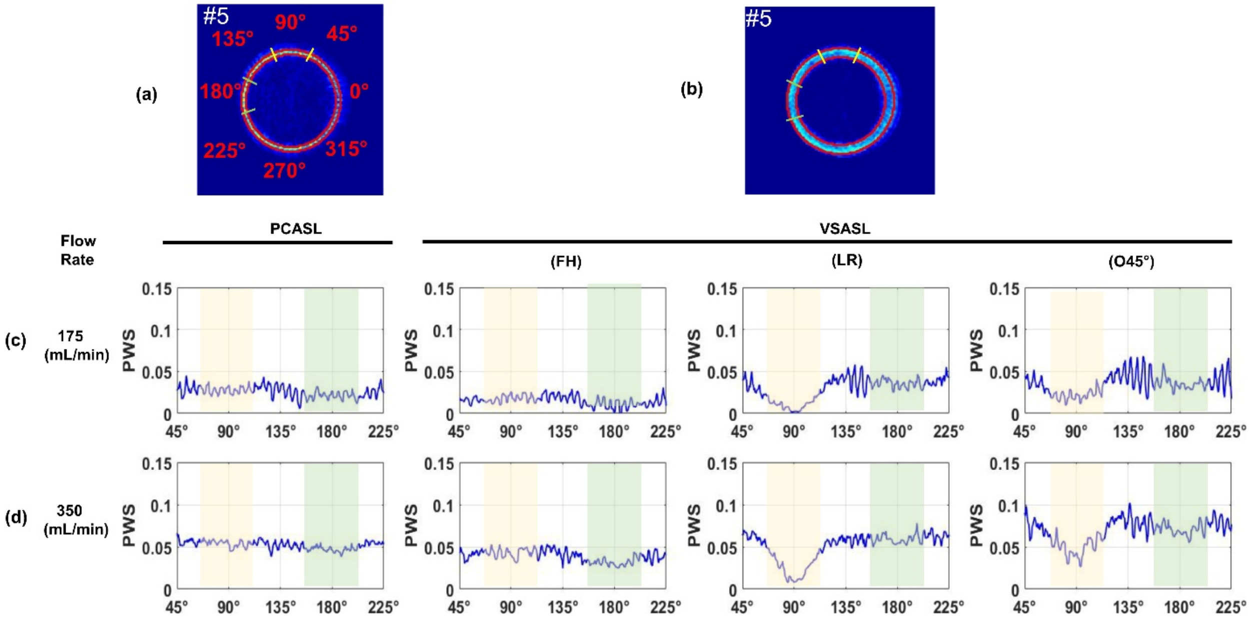

Supplement: 1 — FIGURE S1 Sagittal maximum intensity projection (MIP) of the perfusion phantom obtained by time-of-flight (TOF) (A) and velocity-selective MRA (VSMRA) (B) at a flow rate of 350 mL/min. The yellow lines on TOF indicate the slice location of the MRA images. The yellow lines on VSMRA represent slice locations of the ASL images. (C) Three velocity-encoding directions, foot–head (FH), left–right (LR), and oblique 45° on the coronal plane (O45°), were used in VSMRA and velocity-selective arterial spin labeling (VSASL) scans in this study with the same cut-off velocity FIGURE S2 The signal course within a ring region of interest (ROI) as a function of rotation angles is displayed for TOF and VSMRA with three velocity-encoding directions (A) at 175 mL/min (B) and 350 mL/min (C) flow rates. A, The ring ROI is enclosed by two red circles in the MRA image of slice 29, labeled every 45° around the disk. The left and top sectors were marked with green and yellow bars at the ring ROI (A) and shaded with the same colors at the signal course under each MRA scan (B,C) FIGURE S3 Difference images of PCASL and FH-encoded VSASL at slice 4 with and without the crusher module at 175 mL/min (A) and 350 mL/min (B) FIGURE S4 The perfusion-weighted signal (PWS: difference signal normalized by the SIPD) within a ring ROI as a function of rotation angles is displayed for PCASL and VSASL with three velocity-encoding directions at 175 mL/min (A,C) and 350 mL/min (B,D) flow rates. A,B, The ring ROIs are enclosed by two red circles in the ASL images of slice 5 for each flow rate, respectively. The left and top sectors are marked with green and yellow bars at the ring ROIs (A,B) and shaded with the same colors at the signal course under each ASL scan (C,D) [file NIHMS1779678-supplement-1.pdf]
